# CU-BEMS, smart building electricity consumption and indoor environmental sensor datasets

**DOI:** 10.1038/s41597-020-00582-3

**Published:** 2020-07-20

**Authors:** Manisa Pipattanasomporn, Gopal Chitalia, Jitkomut Songsiri, Chaodit Aswakul, Wanchalerm Pora, Surapong Suwankawin, Kulyos Audomvongseree, Naebboon Hoonchareon

**Affiliations:** 1 Smart Grid Research Unit, Department of Electrical Engineering, Faculty of Engineering, Chulalongkorn University, Bangkok, Thailand; 2 Virginia Tech Advanced Research Institute, Arlington, VA, USA; 3 International Institute of Information Technology Hyderabad, Hyderabad, India; 4 Wireless Network & Future Internet Research Unit, Department of Electrical Engineering, Faculty of Engineering, Chulalongkorn University, Bangkok, Thailand; 5 Energy Research Institute, Chulalongkorn University, Bangkok, Thailand

**Keywords:** Energy management, Energy efficiency, Energy and behaviour

## Abstract

This paper describes the release of the detailed building operation data, including electricity consumption and indoor environmental measurements, of the seven-story 11,700-*m*^2^ office building located in Bangkok, Thailand. The electricity consumption data (kW) are that of individual air conditioning units, lighting, and plug loads in each of the 33 zones of the building. The indoor environmental sensor data comprise temperature (°C), relative humidity (%), and ambient light (lux) measurements of the same zones. The entire datasets are available at one-minute intervals for the period of 18 months from July 1, 2018, to December 31, 2019. Such datasets can be used to support a wide range of applications, such as zone-level, floor-level, and building-level load forecasting, indoor thermal model development, validation of building simulation models, development of demand response algorithms by load type, anomaly detection methods, and reinforcement learning algorithms for control of multiple AC units.

## Abstract (summary)

**Table:** 

Measurement(s)	electrical energy • temperature of air • humidity • visible spectrum radiation
Technology Type(s)	Gauge or Meter Device • Sensor Device
Factor Type(s)	floor • date/time
Sample Characteristic - Environment	building

Machine-accessible metadata file describing the reported data: 10.6084/m9.figshare.12527219

## Background & Summary

The global energy consumption of the building sector which includes both commercial and residential buildings is approximately 20%^1^. With the rapid increase in population as well as economic growth, energy consumption in buildings is projected to increase at the rate of 1.3% per year from 2018 to 2050^2^. With the building sector accounting for one-third of greenhouse gases, two-thirds of halo-carbon and approximately 25–33% of black carbon emissions^3^, this growing energy demand has raised significant concerns worldwide of its negative impact on the environment. Currently available technologies can help reduce electricity consumption in buildings by approximately 30 to 80%^4^. In order to meet the rising electricity demand, an efficient and cost effective operation is needed. Buildings are increasingly equipped with building automation systems (BAS) and smart meters which gather load and other related data at a granular level. And, with such a large amount of building-level data, this has paved ways for data-driven approaches, i.e., statistical/machine learning related approaches instead of the traditional physics based approaches. However, it is very important to have good quality building data for research related to data-driven approaches.

In building electricity research, publicly available datasets are from both commercial and residential buildings.

In the residential space, several public datasets are available at various time resolutions. Typically, one-minute to one-hour resolution data are beneficial for the identification of peak demand reduction opportunities and understanding building electricity characteristics. These are: the UMass smart datasets^5^; the UCI dataset^6^; the Almanac dataset^7,8^; the programmable thermostat data^9^; and the Household Electricity Survey (HES) dataset^10^. The UMass datasets^5^ provide electricity consumption data of 400+ homes at one minute intervals. Potential applications include home energy efficiency improvement, operating cost optimization, peak demand flattening and renewable energy prediction. The UCI dataset^6^ provides electrical consumption data of a house for the period of four years at one minute intervals. It also consists of various sub-metering values, as well as different electrical quantities. The Almanac datasets^7,8^ provide measurements of electricity, natural gas and water at one-minute intervals for the period of around two years. The programmable thermostat dataset^9^ contains relative humidity, temperature and HVAC system state data at 10-minute intervals for 79 apartments. The HES dataset^10^ contains electricity consumption data at an appliance level from 2010 to 2011 for 250 homes in the UK.

Higher resolution data, *i*.*e*., one-second or less, are also available for non-intrusive load monitoring and occupancy detection research. These are such as the PECAN street project dataset^11^, the tracebase dataset^12,13^, the ECO dataset (Electricity Consumption & Occupancy)^14^, Dutch Residential Energy Dataset (DRED)^15,16^, the Reference Energy Disaggregation Dataset (REDD)^17,18^, the BLUED dataset^19,20^, the UK-DALE dataset^21^, the ENERTALK dataset^22^, and several others^23^. The PECAN dataset^11^ has whole-home electricity measurements from 40 homes, including solar generation, electric vehicle (EV) charging, HVAC, major appliances and other in-home circuits, for one year at one-second intervals. This enables remote diagnosis of HVAC systems, solar panels, EV charging systems, and also some household appliances. The tracebase dataset^12,13^ contains power consumption of a range of electrical appliances at one-second intervals in Darmstadt, Germany in 2012 and Sydney, Australia in 2013. The ECO dataset^14^ contains electricity consumption and occupancy data of six Swiss households during the period of eight months, which can be useful for non-intrusive load monitoring and occupancy detection research. The DRED dataset^15,16^ contains details of various parameters of a single household, like occupancy status, ambient conditions, and electricity data for approximately around six months. The REDD dataset^17,18^ contains power consumption data of six households for several weeks, some of which include high-frequency voltage/current data. Current and voltage measurements sampled at 12 kHz are available in the BLUED dataset^19,20^ for a single-family home located in the U.S.. The whole-house electricity consumption data are recorded in the UK-DALE dataset^21^ at the sampling rate of 16 kHz. The whole-house and individual-appliance consumption data are available in the ENERTALK dataset^22^ sampled at 15 Hz.

In commercial building research, most of the available data are building-level electricity consumption data, which can be useful for data analytic and load forecasting. These are such as: the Building Data Genome Project^24^, which provides public datasets from 507 non-residential building electrical meters at one-hour intervals; the ENERNOC dataset^25^, which contains five-minute resolution power consumption data of 100 buildings; the electricity and gas consumption dataset from Lawrence Berkeley National Laboratory (LBNL) Building 74^26^; as well as the 15-minute electricity consumption and outdoor air temperature data for 11 commercial buildings (office/retail)^27^. Some higher resolution data are also available for energy disaggregation research. These are: BERDS (BERkeley EneRgy Disaggregation Dataset)^28^, which includes energy consumption and outdoor temperature data of a building at 20 second intervals; BLOND (a building-level office environment dataset of typical electrical appliances)^29^, which provides whole building energy measurements and appliance-level energy consumption at high sampling rate; and COMBED (the Commercial Building Energy Dataset)^30^, which provides 30-second data across 200 smart meters on a university campus for one month. OpenEI^31^ hosts one-year occupant behavior/environmental data for a medium U.S. office. The data are available for the period of one year, which have been used to track human-building interaction^32^. Building fault detection data^33^ have recently been made available to benchmark the performance of fault detection and diagnostic algorithms.

Additionally, a series of datasets^34^ that relate energy usage in buildings and occupant behaviors are also available. Authors in^35^ provide data of 24 U.S. office occupants, including their thermal comfort, preference, behavioral information, and environmental conditions for one year. Authors in^36^ offer data related to outdoor and indoor environment, as well as energy and occupant behavior collected from 17 cell offices over the period of four years. Authors in^37^ provide data on energy use and occupant behavior recorded from six net-zero energy senior housing units over the period of nine months. And, authors in^38^ provide data on room-level occupant counts and environmental data of three rooms in a building recorded over the period of 44 days.

The uniqueness of the CU-BEMS dataset described in this paper is the breakdown of building-level electricity consumption (kW) into each zone and each floor of the building. The CU-BEMS dataset captures the operation of individual AC units, lighting, and plug loads in each zone of the building at one-minute intervals. These are three major loads in commercial buildings. In addition, corresponding indoor environmental sensor data (temperature, humidity, and ambient light) are also measured in each zone at one-minute intervals. Such a detailed dataset enables potential reuse values, which include:

- Load forecasting at the zone level, floor level, and building level,
- Development and validation of buildings simulation models (electricity consumption and thermal model),
- Development of methods for coordinated control of air conditioning units to reduce the peak demand,
- Development of methods for controlling AC, lighting and plug loads in buildings,
- Anomaly detection of malfunctioned AC and sensors,
- Building-level data analytics.

## Methods

**Fig. 1:**
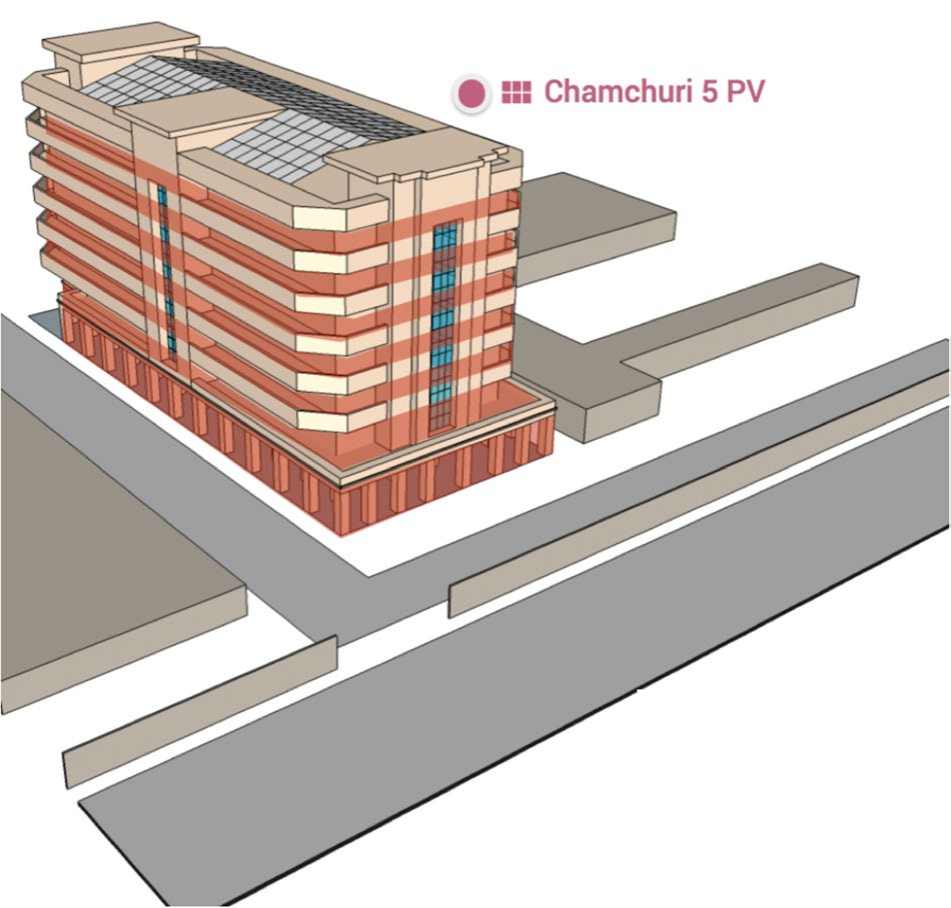
3D visualization of the seven-story academic office building.

In mid-2018, CU-BEMS –the building energy management system, developed at Chulalongkorn University using an open standard IEEE1888, was installed at the seven-story academic office building located at Chulalongkorn University. The building has an area of around 11,700 square meters (126,000 sqft) with a peak load of about 700 kW. The 3D drawing of this building is shown in Fig. 1.

The overall CU-BEMS system comprises Energy Monitoring Units (EMU), digital meters, multi-sensors, gateways and a CU-BEMS server. EMUs, multi-sensors, gateways and the server have been developed in house. Each CU-BEMS building block is explained below.

### Energy Monitoring Unit (EMU)

An EMU is a communicating electrical meter that can measure power consumption of up to 36 circuits and communicate via Ethernet LAN with Modbus protocol. An EMU comprises potential transformers, a microcontroller module and an Ethernet-based communication module. An EMU can connect to up to 36 external current transformers (CT, rating up to 60 Ampere). Based on current and voltage readings, the built-in microcontroller unit calculates power consumption (Watts). Then, the Ethernet module transfers the calculated electricity consumption to the CU-BEMS server using an open standard IEEE 1888 protocol. An EMU has been designed to store data locally during a communication failure, and transmit the data to the server once the communication network comes back. Each EMU has been designed to have class 2 accuracy according to IEC/AS Standard 62053-11. Note that each CT used is of split core type, Heyi KCT-10 (accuracy class 1.0, i.e., typical current error of 1%). To make sure that all readings are not biased, each CT has been calibrated before the installation. For clarity, the manual of the off-the-shelf CT has been uploaded on figshare^39^.

### Digital meter

Each digital meter used is a commercial off-the-shelf product (Siemens SENTRON PAC3100), which provides basic metering and monitoring applications. It provides open communications using Modbus RTU over RS485 interface. It measures current, voltage, and provides real, reactive power measurements, meeting ANSI C12.16 (accuracy class 1.0, i.e., typical error of 1%) specification for revenue meters. For clarity, the manual of the off-the-shelf digital meter has been uploaded on figshare^39^.

### Multi-sensors

Multi-sensors have also been developed in house at the university. It has been designed to measure temperature (0 °C − 90 °C ± 0.4 °C), humidity (0–100%*RH* ± 2%*RH*) and ambient light (0.11 − 10000*lux*). Hence, it comprises temperature, humidity and ambient light sensors, as well as a Wi-Fi communication module.

### Gateway

CU-BEMS gateways have been developed in house to gather data from multi-sensors. Each gateway comprises a microprocessor and an Ethernet module. It has been designed to collect data at one-minute intervals. Table 1 summarizes CU-BEMS hardware deployment.

**Table 1:** CU-BEMS Hardware: the number of deployed units.

No	EMU	Digital Meter	Multi-sensor	Gateway
Floor1	3	1	—	1
Floor2	3	4	4	1
Floor3	3	5	4	1
Floor4	3	5	4	1
Floor5	3	5	4	1
Floor6	3	5	4	1
Floor7	3	5	4	1
**Total**	**21**	**30**	**24**	**7**

**Fig. 2:**
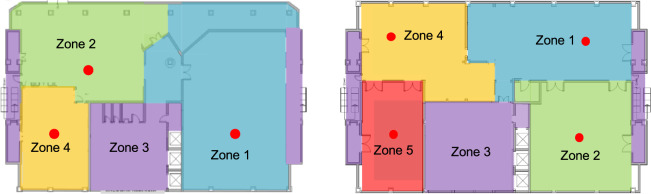
Floor plans on Floors 1–2 (left) and Floors 3–7 (right). Red dots illustrate the approximate locations of multi-sensors on each floor. Note that Floor 1 has no sensor.

The overall CU-BEMS deployment at Chamchuri 5 building comprises the installation of: 21 EMUs, 30 digital meters, 24 multi-sensors, and 7 gateways. Each EMU measures the power consumption of individual wall-mounted AC units, *i*.*e*., 1–2 kW, as well as lighting and plug load circuits. Each digital meter is for measuring a large AC compressor, i.e., 20–40 kW unit. Each multi-sensor measures temperature, humidity, and ambient light condition. Floorplans and approximate locations of multi-sensors are depicted in Fig. 2.

**Fig. 3:**
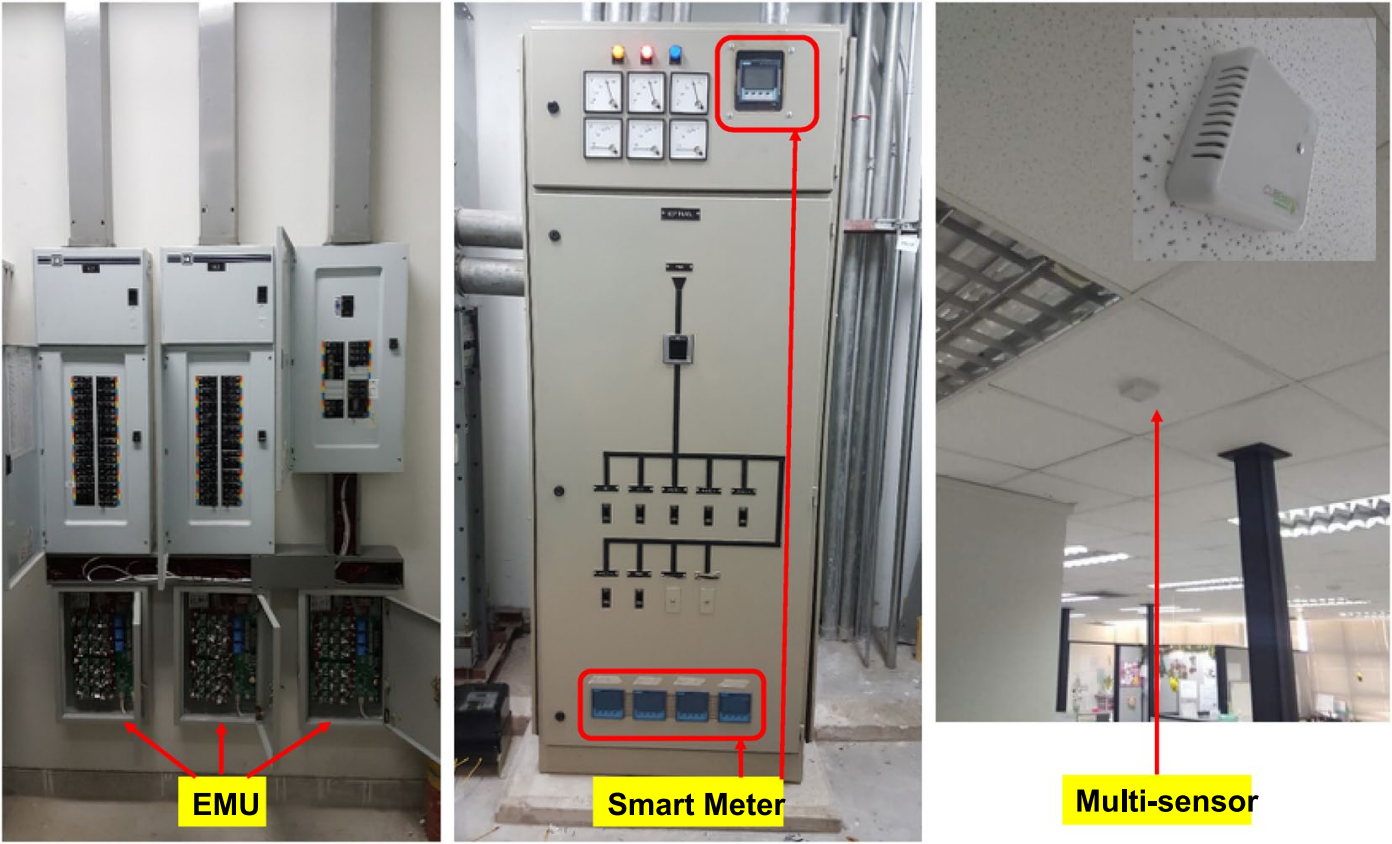
CU-BEMS hardware deployment.

EMUs, smart meters and multi-sensors are depicted in Fig. 3.

**Fig. 4:**
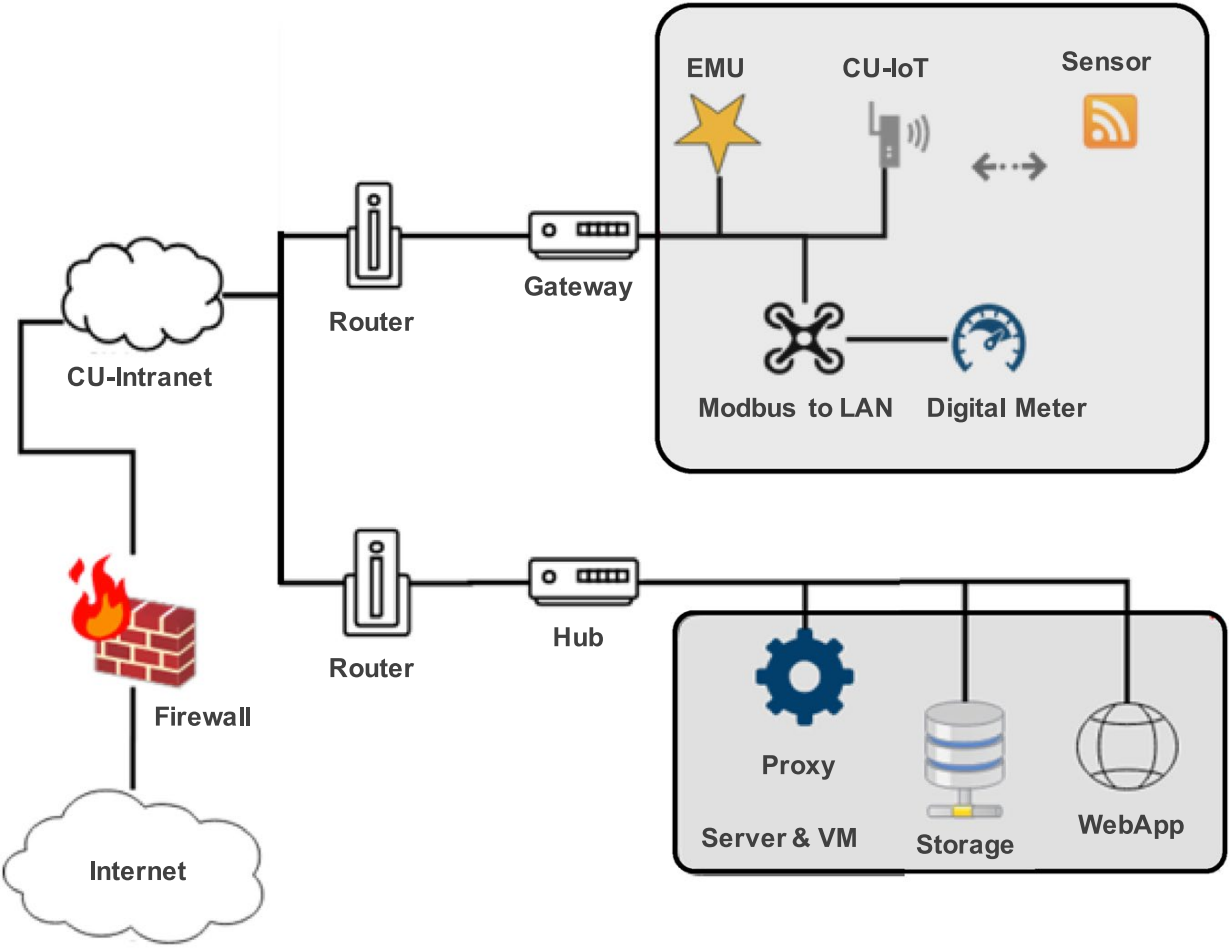
Overall communication architecture.

Each gateway networks all hardware devices on the same floor to the CU-BEMS server. Figure 4 illustrates how all hardware devices are networked together.

As shown in Fig. 4, on each floor, EMUs, digital meters, and sensors communicate with a gateway via Ethernet LAN, Modbus TCP, and Wi-Fi (CU-IoT), respectively. Note that EMU has been developed in house at Chulalongkorn University. Since each EMU needs to transmit electrical readings from 36 channels, it is important to choose a reliable communication technology. Hence, LAN has been chosen to support EMU communications. Digital meters, on the other hand, are commercial off-the-shelf products. Modbus is a popular protocol as it communicates over RS-485, which is noise-tolerance for long distance communications. For multi-sensors, since multi-sensors need to be placed on the ceiling, it is practical to connect the sensors using WiFi. The CU-BEMs server is located in the building, comprising the proxy server, the storage unit, and the web application. The networking of equipment with the server is via the University’s Intranet (CU-Intranet).

## Data Records

The entire datasets are divided into 14 comma-separated value (csv) files according to the floor and year of the data recorded, as summarized in Table 2. This is because CSV files can be easily imported in spreadsheets, or any database or a programming language, making it easier and more organized to work with. Note that one CSV file is provided for each floor of the building. This makes the total of seven CSV files for each year. Since each file does include data of each zone on a single floor, a user has the flexibility to work with any individual zones, which can be extracted (based on the column names) from the CSV files. These data are available for download on figshare^39^.

**Table 2:** CU-BEMS dataset file names.

	Year 2018	Year 2019
Floor1	*2018Floor1*.*csv*	*2019Floor1*.*csv*
Floor2	*2018Floor2*.*csv*	*2019Floor2*.*csv*
Floor3	*2018Floor3*.*csv*	*2019Floor3*.*csv*
Floor4	*2018Floor4*.*csv*	*2019Floor4*.*csv*
Floor5	*2018Floor5*.*csv*	*2019Floor5*.*csv*
Floor6	*2018Floor6*.*csv*	*2019Floor6*.*csv*
Floor7	*2018Floor7*.*csv*	*2019Floor7*.*csv*

Each file combines the measurements available in each zone on the same floor of the building in a particular year. These measurements are summarized in Table 3, which are the electricity consumption (kW) of individual air conditioning (AC) units, lighting loads and plug loads, as well as the environmental sensor data, including indoor temperature (°C), relative humidity (%) and ambient light (lux). Note that the monitored loads do not include the two elevators and emergency exit signs. These loads added up to about 1–2 percent of the total building loads.

**Table 3:** Available measurements and their units of records in CU-BEMS dataset.

File name	Category	Measurement (time-series)	Unit
YFloorX.csv	Electricity consumption data	Individual air conditioning (AC) unit	kW
for X ∈ [1, …, 7]		Lighting load	kW
		Plug load	kW
for Y ∈ [2018, 2019]	Indoor environmental sensor data	Indoor temperature	°C
		Relative humidity	%
		Ambient light	lux

Each of the 2018 data files has 264,960 rows, which indicate one-minute interval data (1,440 data points/day) for 184 days during the second half year of 2018. Each of the 2019 data files has 525,600 rows, which indicate one-minute interval data (1,440 data points/day) for 365 days during the entire year of 2019.

The number of columns is different in each file, depending on the number of data measurements on the floor. Table 4 summarizes the number of measurements available in each zone on each floor of the building.

**Table 4:** The number of electricity consumption and indoor environmental data.

File name	Zone No.	AC	Light	Plug	Sensor	No of Data Columns
Floor1.csv	Zone 1	0	1	0	0	11
	Zone 2	4	1	1	0	
	Zone 3	0	1	1	0	
	Zone 4	0	1	1	0	
Floor2.csv	Zone 1	1	1	1	3	36
	Zone 2	14	1	1	3	
	Zone 3	0	1	1	3	
	Zone 4	1	1	1	3	
Floor3.csv	Zone 1	4	1	1	3	29
	Zone 2	1	1	1	3	
	Zone 3	0	1	1	0	
	Zone 4	1	1	1	3	
	Zone 5	1	1	1	3	
Floor4.csv	Zone 1	4	1	1	3	29
	Zone 2	1	1	1	3	
	Zone 3	0	1	1	0	
	Zone 4	1	1	1	3	
	Zone 5	1	1	1	3	
Floor5.csv	Zone 1	4	1	1	3	29
	Zone 2	1	1	1	3	
	Zone 3	0	1	1	0	
	Zone 4	1	1	1	3	
	Zone 5	1	1	1	3	
Floor6.csv	Zone 1	1	1	1	3	29
	Zone 2	1	1	1	3	
	Zone 3	0	1	1	0	
	Zone 4	4	1	1	3	
	Zone 5	1	1	1	3	
Floor7.csv	Zone 1	4	1	1	3	29
	Zone 2	1	1	1	3	
	Zone 3	0	1	1	0	
	Zone 4	1	1	1	3	
	Zone 5	1	1	1	3	
**TOTAL**	**All zones**	**55**	**33**	**32**	**72**	**192**

For example, the files *2018Floor1*.*csv* and *2019Floor1*.*csv* have 11 data columns, and one timestamp column. These 11 data columns are: Zone 1–Power consumption (kW) of lighting loads (one column); Zone 2–Power consumption (kW) of four individual AC units, one lighting load and one plug load (six columns); Zone 3–Power consumption (kW) of one lighting and one plug loads (two columns); and Zone 4–Power consumption (kW) of lighting and plug loads (two columns). Floor 1 has no sensor.

The files *2018Floor2*.*csv* and *2019Floor2*.*csv* have 36 data columns, which are: Zone 1–Power consumption (kW) of the AC unit, lighting loads and plug loads, as well as indoor temperature (deg C), relative humidity (%) and ambient light condition (lux) measured in this zone (six columns). Zone 2–Power consumption (kW) of 14 individual AC units, lighting loads and plug loads, as well as indoor temperature (deg C), relative humidity (%) and ambient light condition (lux) in this zone (19 columns); Zone 3–Power consumption (kW) of lighting loads and plug loads, and indoor temperature (deg C), relative humidity (%) and ambient light condition (lux) in this zone (five columns); and Zone 4: Power consumption (kW) of the AC unit, lighting loads, plug loads and indoor temperature (deg C), relative humidity (%) and ambient light condition (lux) in this zone (six columns).

For Floor 3 to Floor 7, each floor has five zones. Each zone has one lighting load and one plug load measurements. There are a total of seven AC units and four sensors (each measuring three quantities: temperature, humidity, and ambient light) on each floor. Hence, each file has 29 data columns.

For the entire building, there are power consumption data of 55 individual AC units; power consumption of lighting loads in 33 zones of the building; power consumption of plug loads in 32 zones of the building (Zone 1 on Floor 1 does not have plug load); and temperature, humidity and ambient light readings at 24 locations (72 values) in the building.

## Technical Validation

This section presents the visualization of data to show the quality and technical validity of the dataset, including missing data, histogram, and weekly pattern plots. The missing data plot provides insight into data availability. Data histogram plots help to understand the range of measurements on each floor/zone of the building. Weekly patterns show the relationship between AC operation and indoor temperature/humidity, as well as lighting/plug load operation and indoor ambient light conditions.

### Missing data

#### AC load data

**Fig. 5:**
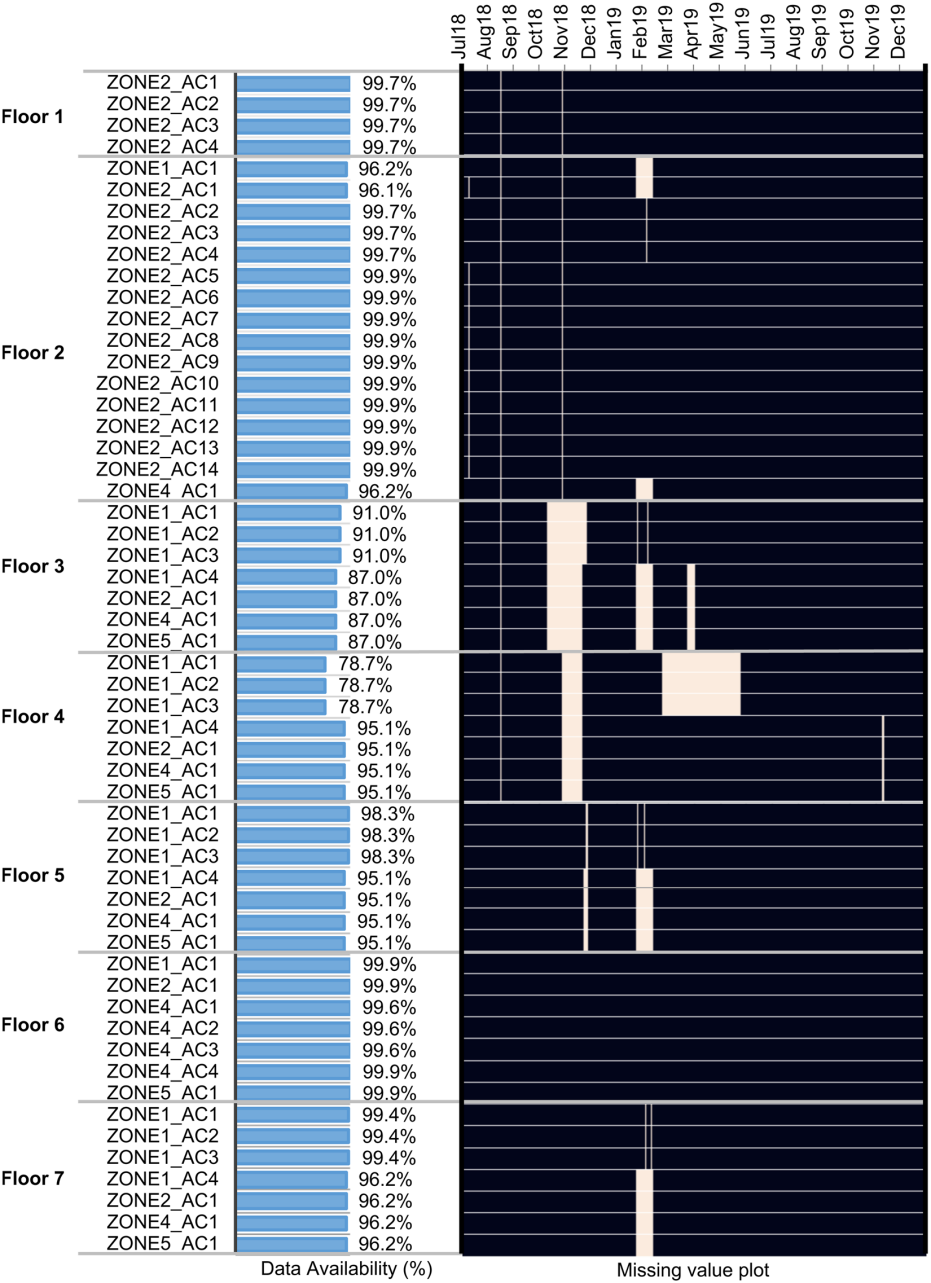
Data availability for **all 55 AC units** from July 2018 to December 2019 (black = data available).

Figure 5 depicts missing power consumption data of all 55 AC units, grouped by floors (1–7) and by zones (1–5). The horizontal bar chart on the left summarizes the percentage of data availability, and the missing data plot is shown on the right from July 2018 to December 2019 (where *white* indicates the missing data). As shown, the majority of the AC units have data availability of at least 95%. The exceptions are for some AC units on Floor 3 and Floor 4, which have around 10–20% of missing data. The reason for missing data was because of the change in IT configurations of the entire floors. Hence, the corresponding gateways could not communicate to the main network during such periods. Note: Only Zone 2 on Floor 1 is air-conditioned. There is no AC in Zone 3 on all floors because Zone 3’s are non air-conditioned staircases.

#### Lighting and plug load data

**Fig. 6:**
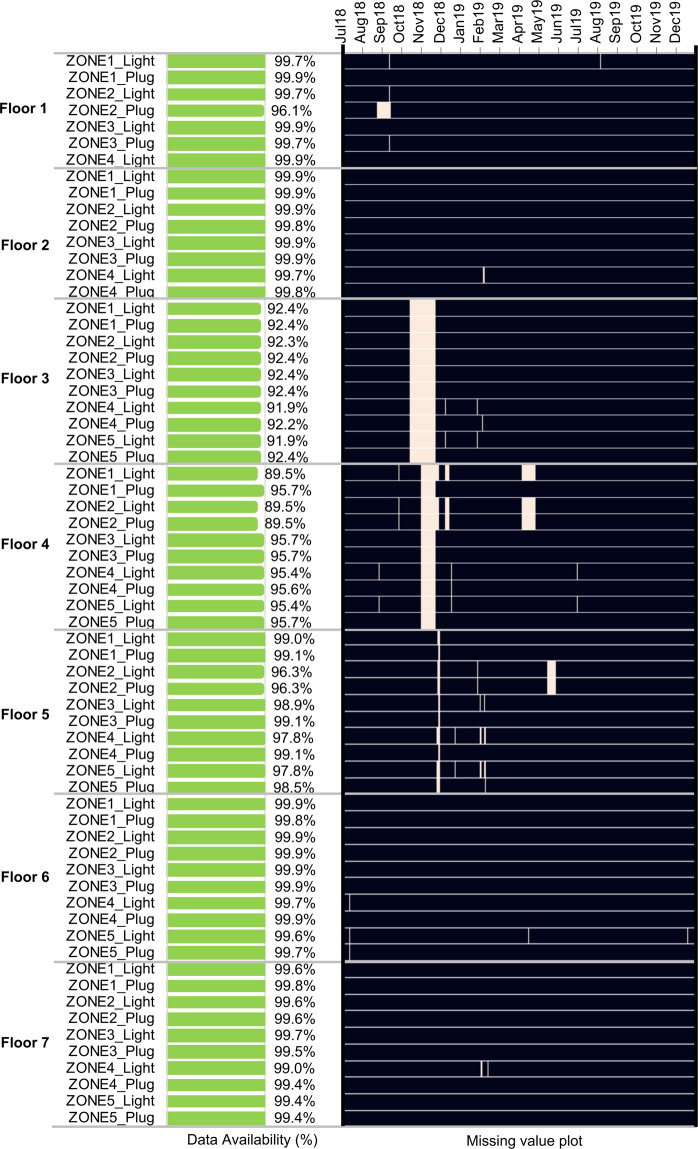
Data availability for **all 33** + **32 lighting and plug loads** from July 2018 to December 2019 (black = data available).

Figure 6 plots the missing power consumption data of all 33 lighting and 32 plug load measurements, grouped by floors and by zones. Similar to those of AC units, the majority of lighting and plug loads have data availability of 95% or higher, except for some lighting and plug loads on Floor 3 and Floor 4, which have around 10% of missing data. Again, the missing data were attributed to the LAN communication network interruption.

#### Sensor data

**Fig. 7:**
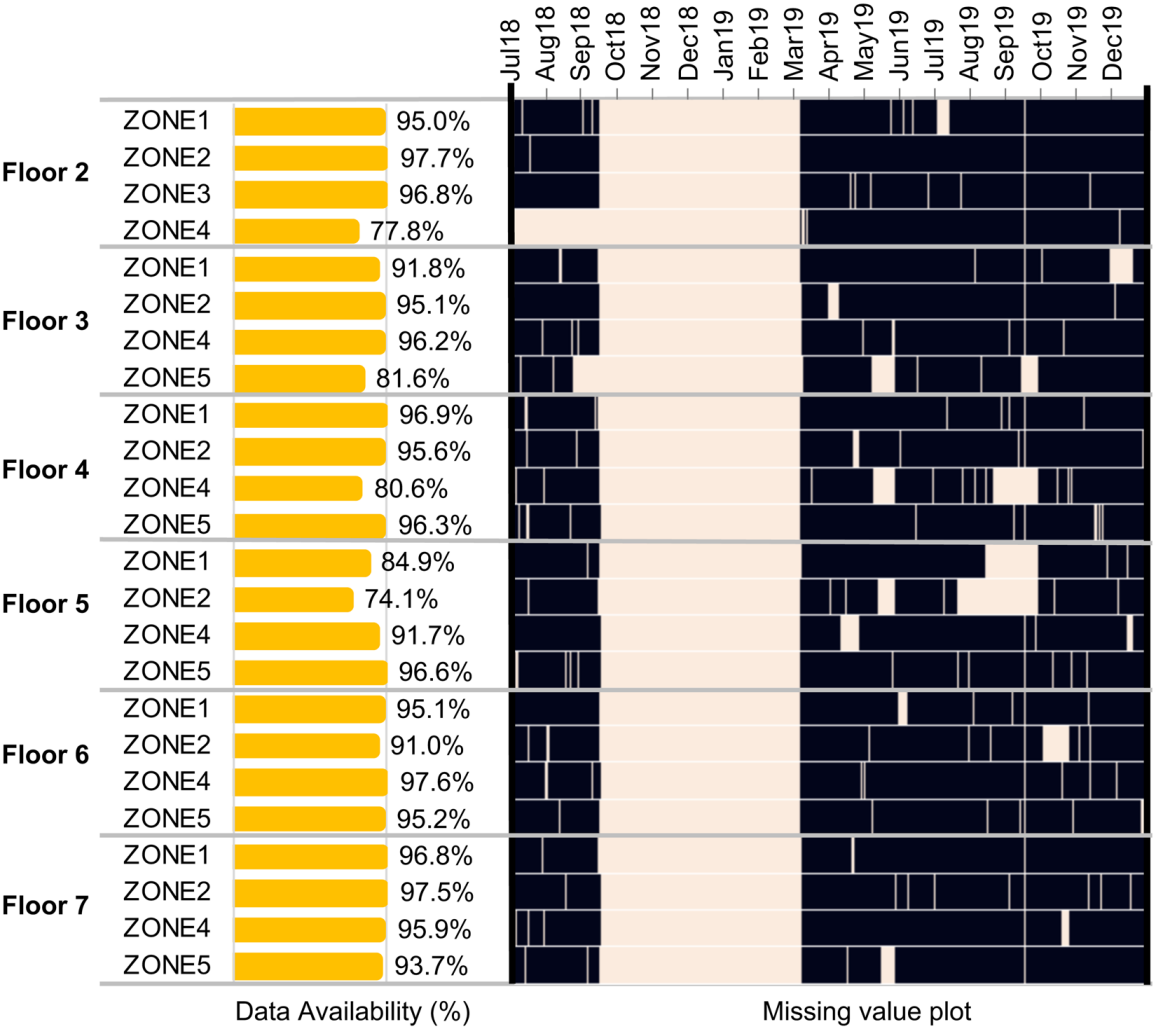
Data availability for **all sensors** from July 2018 to December 2019 (black = data available). Sensors were taken down for maintenance between September 15, 2019 and March 5, 2019. This period was excluded from calculating data availability.

Figure 7 shows the missing sensor data. Note that Floor 1 has no sensor and that there is no sensor in Zone 3 on each floor (except Floor 2) as they are non air-conditioned staircases. During the period between September 15, 2018, and March 5, 2019, the CU-BEMS team took all sensors down for maintenance. Note that: at the beginning of the measurement campaign, sensors were disconnected often and this was deemed because of the firmware. Hence it was necessary to take down the sensors to update the firmware. Since the sensors were taken down for six months, the measurement period hence was extended for an additional six months to compensate for the missing period. Overall, for most of the sensors, data are available more than 95% of the time. There have been sporadic missing data because the sensors communicate via Wi-Fi, and they occasionally are disconnected from the network.

### Data histograms

**Fig. 8:**
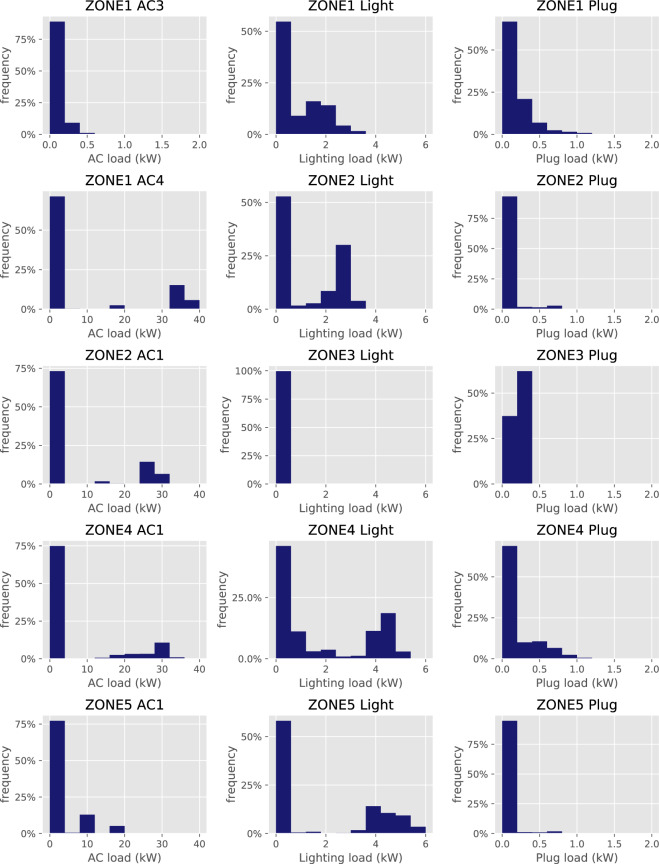
Power consumption histograms of selected AC units (left), lighting (middle) and plug loads (right) on Floor 7, zones 1–5.

Figure 8 depicts the histogram plots, capturing the power consumption of selected AC units, lighting loads, and plug loads on Floor 7. As shown, AC3 in Zone 1 is a window unit, and its power consumption is between 0 and 1 kW. The rests of the AC units shown are larger units with a peak load between 20 and 40 kW. For the lighting loads, the peak loads range from 1 to 6 kW, depending on the zone. Zone 3 has the lowest lighting load consumption as it is a staircase. For the plug loads, their peak loads are between 0.5 and 1.5 kW. Zone 3 again has the lowest plug load consumption for the same reason. Since the power consumption of each device, i.e., AC, lighting and plug loads, seems to coincide with its rating and usage characteristics, the power consumption histograms are deemed to be valid.

**Fig. 9:**
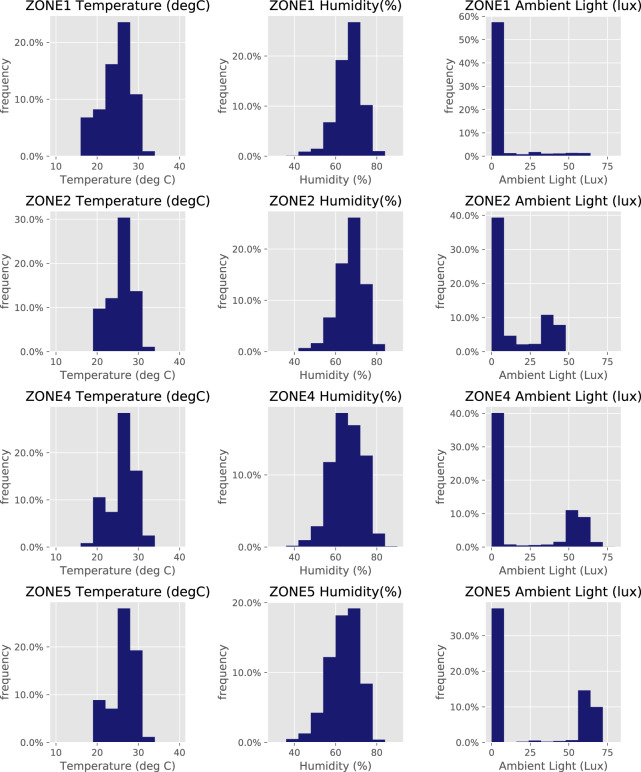
Histograms of indoor temperature (left), humidity (middle) and ambient light (right) on Floor 7, zones 1–5.

Figure 9 illustrates the histogram plots of temperature, humidity, and ambient light on Floor 7. Temperatures on Floor 7 are between 18 to 35 °C and humidity varies from 40 to 80%. This is in line with typical temperature and humidity in Thailand. The ambient light condition varies between 0 and 75 lux, depending on the zone. The readings are also in line with the ambient light conditions in this building. Note that all sensors are attached to the ceiling.

### Weekly patterns

**Fig. 10:**
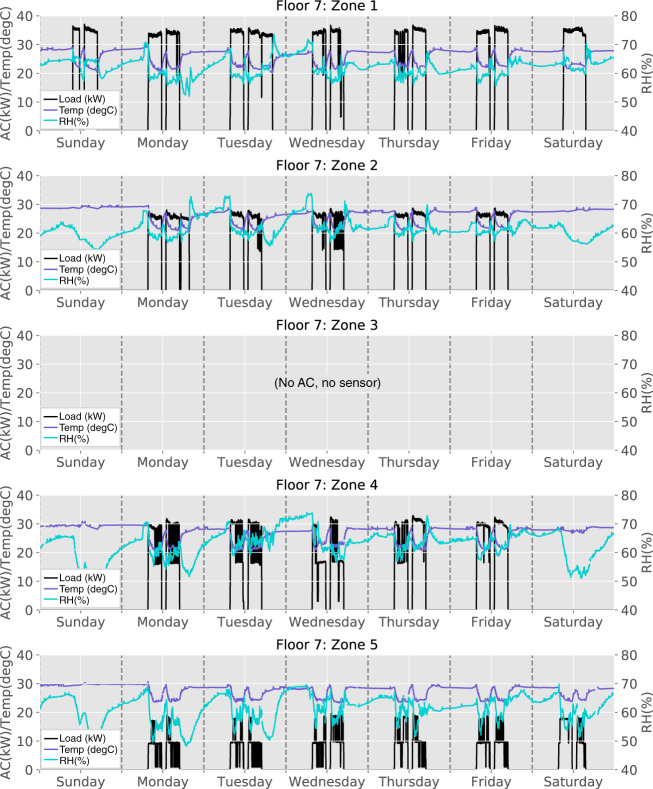
AC power consumption, indoor temperature and humidity during a one-week period on Floor 7, zones 1–5.

Figure 10 shows the electricity consumption (kW) of AC units, indoor temperature (°C), and humidity (%) measurements in each zone on Floor 7, during a period of one week from Sunday to Saturday (August 5–11, 2018). Here, only the consumption of large AC units (20–40 kW) is plotted as they have high influence on indoor temperature and humidity in their respective zone. As expected, when an AC unit operates, the indoor temperature in the same zone drops correspondingly; and when an AC unit is turned off, the indoor temperature rises. Zone 3 is non air-conditioned, and there is no AC, nor sensor data to display.

**Fig. 11:**
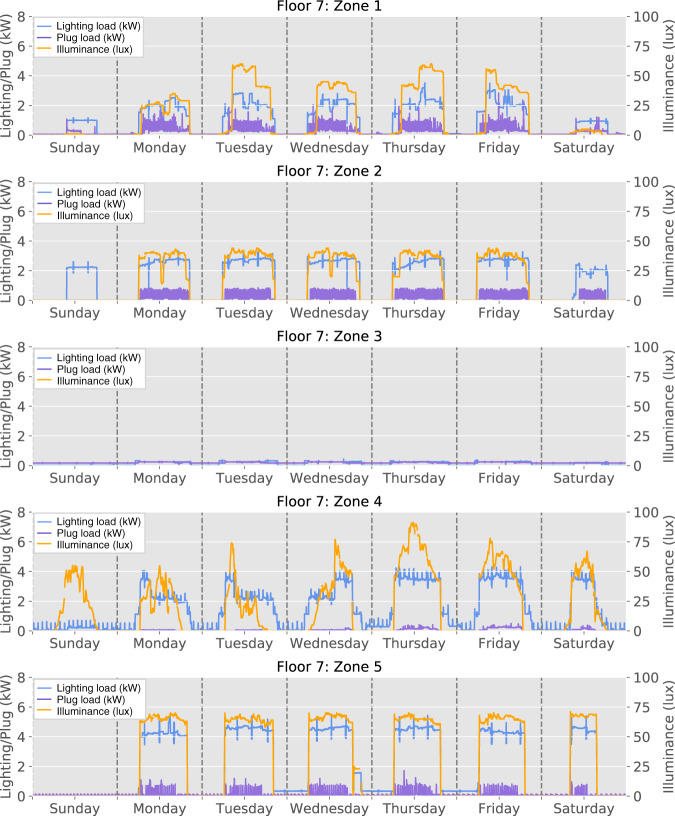
Lighting/plug load consumption and ambient light conditions during a one-week period on Floor 7, zones 1–5.

Figure 11 illustrates the relationship between lighting/plug load consumption (kW) and ambient light measurements (lux) on Floor 7 in each zone, during the same period. As can be observed, when the lights are on (*i*.*e*., the power consumption of lighting load is non-zero), ambient light measurement of the corresponding zone increases. Also, the ambient light measurements in Zone 4 indicate that the sensors can capture natural light. In Zone 2, there is a partition within the zone that influences the amount of light that the sensor can capture. This reflects in the measurements on Sunday and Saturday, when not all lighting loads are turned on.

## Usage Notes

Since each csv file is large (30–80 MB), it is not suitable to be open in Excel. It is suggested to use a programming language, like Python, for data visualization and manipulation. The code to perform data visualization as presented in this paper is available at SGRU github (https://sgrudata.github.io/) under “Sample Data Analytics” tab. Python libraries, such as Numpy, Pandas, Matplotlib and Seaborn, were used.

## Data Availability

The code implementation was done in Python3 using Jupyter notebook. The scripts to perform data pre-processing, technical validation, visualization are available at SGRU github repository (https://nbviewer.jupyter.org/github/mpipatta/mpipatta.github.io/blob/master/CHAM5.ipynb).
